# A two-step registration-classification approach to automated segmentation of multimodal images for high-throughput greenhouse plant phenotyping

**DOI:** 10.1186/s13007-020-00637-x

**Published:** 2020-07-09

**Authors:** Michael Henke, Astrid Junker, Kerstin Neumann, Thomas Altmann, Evgeny Gladilin

**Affiliations:** Leibniz Institute of Plant Genetics and Crop Plant Research (IPK), OT Gatersleben, Corrensstrasse 3, 06466 Seeland, Germany

**Keywords:** Greenhouse plant phenotyping, Visible light imaging, Fluorescence imaging, Multimodal image alignment, Supervised image segmentation, Machine learning

## Abstract

**Background:**

Automated segmentation of large amount of image data is one of the major bottlenecks in high-throughput plant phenotyping. Dynamic optical appearance of developing plants, inhomogeneous scene illumination, shadows and reflections in plant and background regions complicate automated segmentation of unimodal plant images. To overcome the problem of ambiguous color information in unimodal data, images of different modalities can be combined to a virtual multispectral cube. However, due to motion artefacts caused by the relocation of plants between photochambers the alignment of multimodal images is often compromised by blurring artifacts.

**Results:**

Here, we present an approach to automated segmentation of greenhouse plant images which is based on co-registration of fluorescence (FLU) and of visible light (VIS) camera images followed by subsequent separation of plant and marginal background regions using different species- and camera view-tailored classification models. Our experimental results including a direct comparison with manually segmented ground truth data show that images of different plant types acquired at different developmental stages from different camera views can be automatically segmented with the average accuracy of $93\%$ ($SD=5\%$) using our two-step registration-classification approach.

**Conclusion:**

Automated segmentation of arbitrary greenhouse images exhibiting highly variable optical plant and background appearance represents a challenging task to data classification techniques that rely on detection of invariances. To overcome the limitation of unimodal image analysis, a two-step registration-classification approach to combined analysis of fluorescent and visible light images was developed. Our experimental results show that this algorithmic approach enables accurate segmentation of different FLU/VIS plant images suitable for application in fully automated high-throughput manner.

## Background

**Fig. 1:**
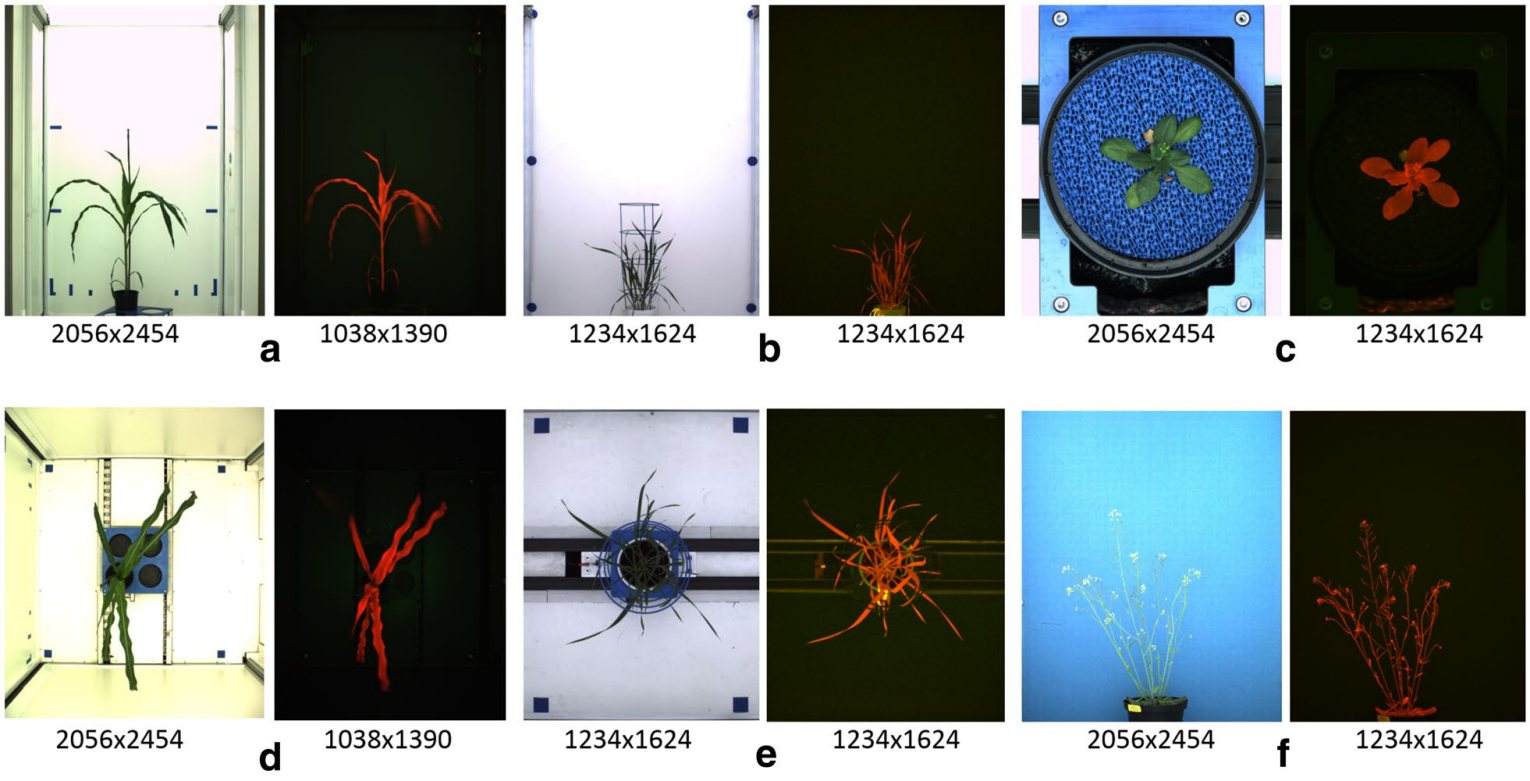
Examples of FLU/VIS images of maize (**a**, **d**), wheat (**b**, **e**) and arabidopsis (**c**, **f**) shoots taken at different phenotyping facilities with different camera views (side/top) and spatial resolutions

In the last two decades, high-throughput greenhouse phenotyping became the method of choice for quantitative assessment of plant morphology, development and function. High-throughput screening platforms such as LemnaTec-Scanalyzer3D (LemnaTec GmbH, Aachen, Germany) enable, depending on the configuration, the acquisition of thousands of fluorescence (FLU), visible light (VIS), near-infrared (NIR) images that have to be processed and analyzed in an automated manner. The first essential step of plant image analysis, which determines the quality of all subsequently derived phenotypic traits, consists of robust and accurate segmentation (i.e. spatial localization) of plant structures. A straightforward segmentation of optically heterogeneous and noisy greenhouse images is, however, hampered by a combination of several natural and technical factors, including variable optical appearance of developing plants, inhomogeneous scene illumination, occlusions, shadows and reflections in for- and background image regions, see Fig. 1. Consequently, same or similar colors may occur in plant and non-plant image regions, which makes application of simple color-thresholding techniques improper. The principal difficulty of accurate segmentation of optically complex and dynamic greenhouse images was identified as the major bottleneck of high-throughput plant phenotyping [1].

State of the art approaches to segmentation of plant images include

- Application of saliency approaches based on certain assumptions about image structure [2, 3], for example, that majority of image pixels belong to plant-free background region,
- Construction of color-distance maps followed by their subsequent thresholding or clustering [4, 5],
- Application of supervised and unsupervised classification and machine learning models [6, 7],
- Co-registration of different image modalities, e.g., visible light (VIS) and infrared (IR) images [8], high-contrast fluorescence (FLU) and low-contrast visible light (VIS) or near-infrared (NIR) images [9].

Unfortunately, the prerequisites for saliency approaches is not always given. Sometimes, plant structures overgrow the optical field so that majority of pixels cannot be considered as background. Efficient and straightforward in algorithmic implementation color-distance methods become less reliable in presence of shadows and illumination changes. In such cases, reference images (i.e. background illumination without any plants) may substantially deviate from the background regions of plant-containing images. Especially, adult plants with large and/or many leaves throw large shadows that alter original colors and intensity distribution of background regions and low-lying leaves.

Supervised machine and, in particular, deep learning techniques are nowadays successfully applied for plant image processing and analysis [10]. However, optical appearance of diverse plant types under different experimental conditions exhibits large variability which requires substantial efforts for generation of reliable ground truth data. Especially, advanced deep learning methods are known to require a large amount of representative, manually annotated images that may reasonably be generated for a one or few simple model species with the help of unskilled contributors [11], but can hardly be extended to many other crop plant species imaged in different camera views, with different camera modalities, at different developmental stages. Promising statistical approaches to unsupervised segmentation of VIS plant images using were presented in [12, 13]. Further investigation are, however, required to assess their robustness and efficiency by application to large amount of heterogeneous greenhouse plant images.

**Fig. 2:**
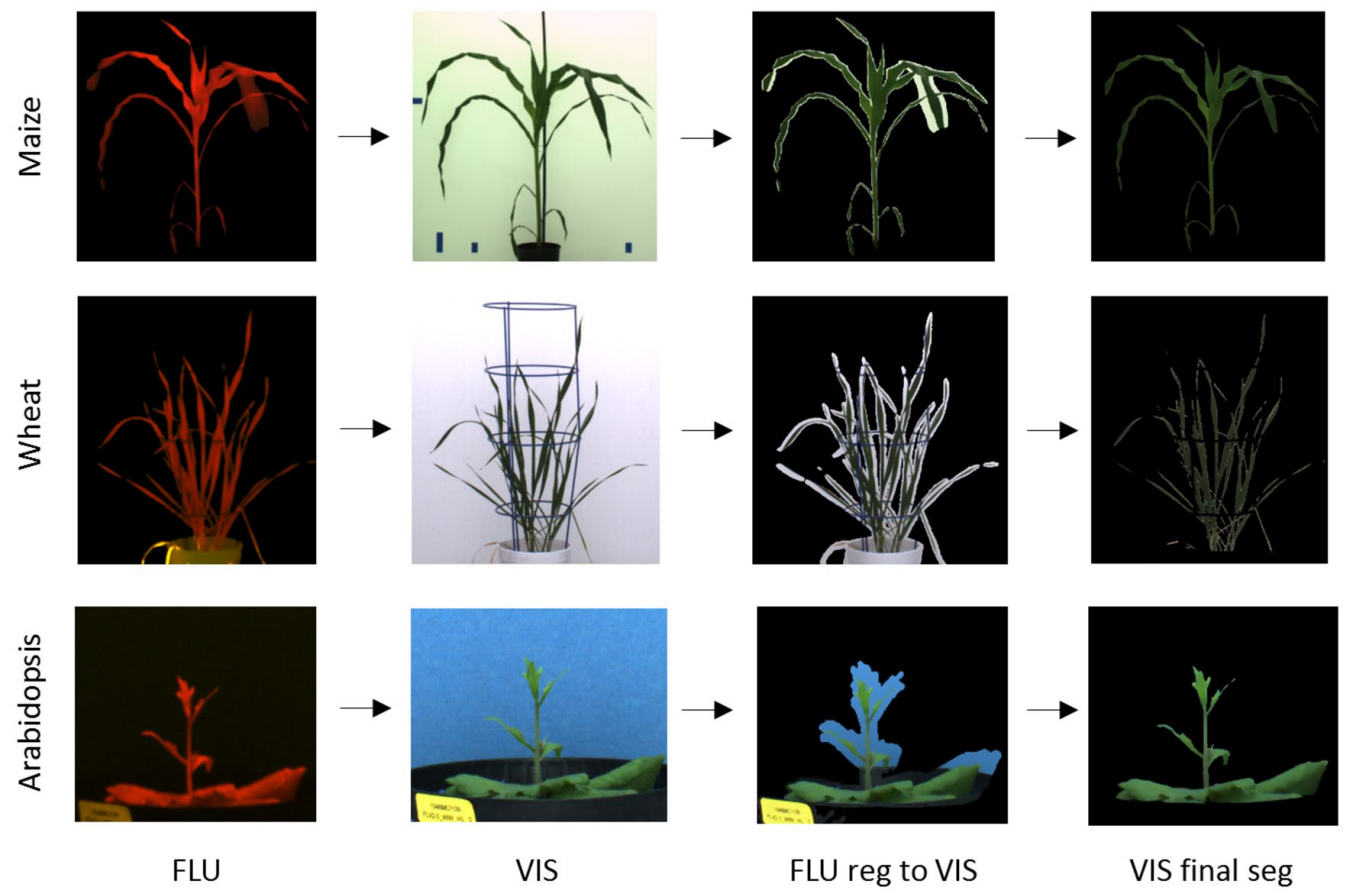
The principle scheme of the registration-classification approach to shoot image segmentation. From left to right: higher contrast FLU images are automatically segmented and registered to VIS images. Due to motion artefacts, FLU/VIS co-registration is not exact which results in inclusion of marginal background regions. For separation of plant and marginal background regions supervised classification models trained for particular a case-scenario including camera view (top/side), plant type (arabidopsis, wheat, maize), developmental stages (juvenile, mid-stage, adult shoots) are applied

To overcome the above limitations of unimodal image analysis, combination of images of different modalities, for example, high-contrast FLU and low-contrast VIS images, was suggested in our previous works [9]. Once aligned, the binary mask of segmented FLU image can be applied for extraction of target plant regions in structurally more complex and difficult VIS images. However, due to unavoidable inertial motion of plant leaves by relocation of plants from one photochamber to another, images of different modalities may exhibit relative non-uniform motion which leads to locally inexact co-registration and inclusion of marginal background regions, see Fig. 2. In this work, we present a two-step algorithmic approach which combines multimodal image registration with subsequent detection and elimination of marginal background regions using supervised classification models of optical plant and background appearance in extended color spaces. Our experimental results show that combination of spatial and color information from multimodal image co-registration and color classification enables an accurate and robust segmentation of plant images in context of high-throughput greenhouse plant phenotyping. Precompiled executables of our multimodal plant image registration-classification-segmentation pipeline suitable for a straightforward command-line script application accompany this work.

## Methods

### Image data acquisition and pre-processing

Visible light (VIS) and fluorescence (FLU) top-/side-view images of developing arabidopsis, wheat and maize shoots were acquired from high-throughput measurements over more than two weeks using three different LemnaTec-Scanalyzer3D platforms for high-throughput phenotypic of small (e.g., arabidopsis), mid-size (e.g., wheat), and large (e.g., maize) plants (LemnaTec GmbH, Aachen, Germany), see Table 1. For a detailed specification of VIS/FLU camera sensors and filters we refer to our previous publication [14]. For quantification of accuracy of image segmentation algorithms, images were segmented manually using our in-house kmSeg tool, which relies on efficient annotation of automatically pre-segmented image regions to plant or non-plant binary categories using k-means clustering of Eigen-colors [15].

**Table 1:** An overview of image data used in this study including three different experiments of three different species, each taken in visible light and fluorescence, obtained by three different LemnaTec high-throughput phenotyping facilities for large, intermediate size and small plants at the IPK Gatersleben

Plants/views	# plants	# days	# angles	# FLU/VIS pairs	VIS size	FLU size
Arab.T./top	4	20	1	80	2056 × 2454	1234 × 1624
Wheat/side	4	47	3	564	1234 × 1624	1234 × 1624
Maize/side	6	22	4	526	2056 × 2454	1038 × 1390

### Distance-based pre-segmentation

Our previous investigations have shown that multimodal image registration is sensitive to structural differences between images such as background image gradient, shadows and reflections [9]. In order to improve robustness of multimodal image co-registration, VIS and FLU images are automatically pre-segmented using the following basic steps:

- Computation of the Euclidean distance in the RGB color space between the reference (empty background) and the plant containing image,
- Clustering of the distance image into a predefined number (N) of clusters using the fast equidistant k-means algorithm (in this work N = 25 was used to separate plant from noisy background regions),
- Calculation of z-scores between color distributions of background and plant-containing images for all N k-means clusters,
- Selection of k-means clusters with z-score values of plant-background color distributions exceeding a certain threshold value (in this work the z-score threshold $\text {tsh}=5$ was used).

**Fig. 3:**
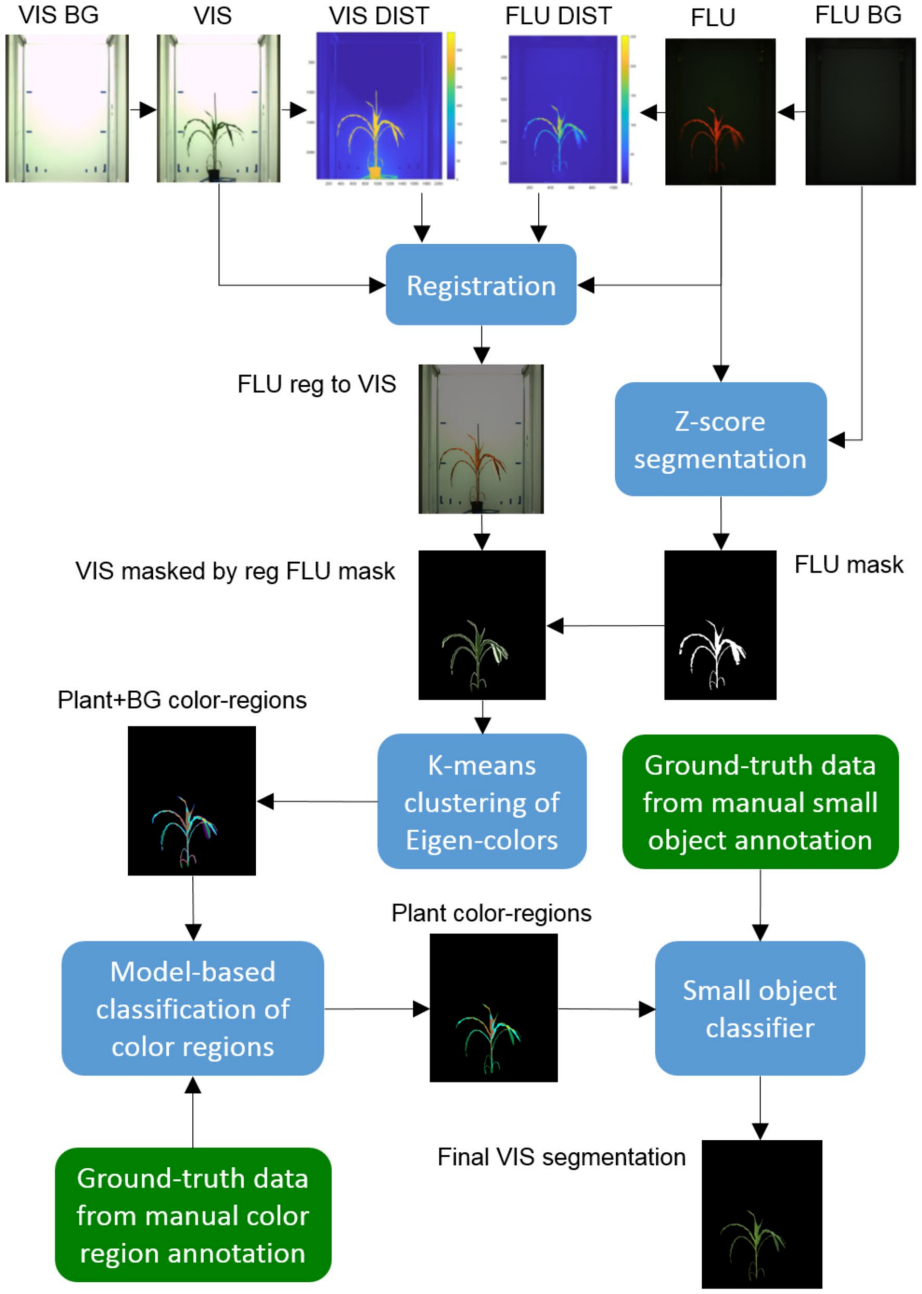
The pipeline of data processing for the registration-classification based plant multimodal plant image segmentation. From top to bottom: color distance maps between reference (empty background) and plant containing FLU and VIS images are computed and subsequently used for co-registration of FLU and VIS images. High contrast FLU images are automatically segmented using the fixed z-score threshold for local color distance between the reference (background) and plant-containing images. Binary mask of the registered FLU image is applied to detect plant structures in the VIS image. Due to motion artefacts regions of the VIS image overlaid by registered FLU mask may contain plant as well as marginal background structures. Unsupervised k-means clustering of Eigen-colors is applied to generate a compact representation of pre-segmented VIS images by a small number (10–30) of color-region centroids. Pre-trained classification models of plant/background color-regions and small object filters are applied to differentiate between plant and non-plant structures

Pre-segmentation performed using this approach enables elimination of background regions that would otherwise irritate registration algorithms. As a result of pre-segmentation one obtains a pair of almost ideally segmented FLU and roughly cleaned VIS images that exhibit structural features (e.g., shape contours) required for detection of FLU/VIS images similarities and their automated alignment, see Fig. 3.

### FLU/VIS image co-registration

Pre-segmented FLU and VIS images are automatically aligned using the iterative image co-registration scheme as described in [9]. The transformation matrix obtained from registration of pre-segmented FLU/VIS images is then used to mask the plant regions of VIS image corresponding to the automatically segmented and registered FLU binary mask, see Fig. 3.

### Transformation of RGB images to Eigen-color space

**Fig. 4:**
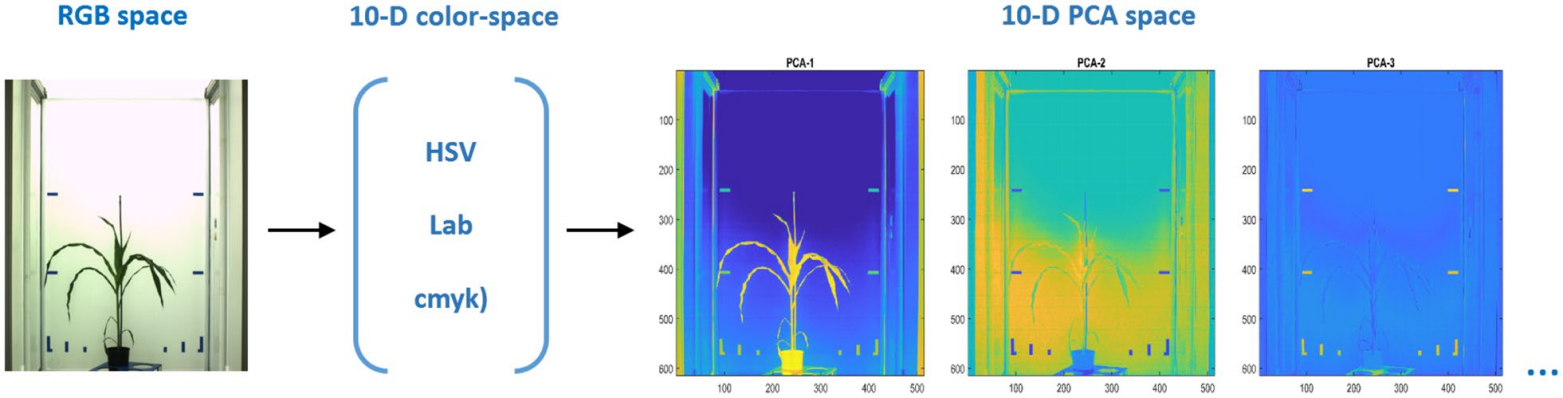
Example of color space transformation of a RGB visible light image of a maize shoot to 10-dimensional (HSV + Lab + cmyk) and Eigen-color spaces. In the figure, only first three out of ten Eigen-color components are shown

FLU and VIS images are transformed from RGB to HSV (3D), Lab (3D) and CMYK (4D) color spaces and subsequently merged to a 10 dimensional (i.e. $3+3+4$) color space representation. To improve topological separability of color clusters, principal component analysis (PCA) of 10 dimensional color space is performed to obtain ’Eigen-color’ image representation, see Fig. 4.

### Data reduction using k-means clustering

Pixel-wise description of plant and background image structures leads to extremely large data, e.g., a 2592x3872 RGB image has $3e+7$ data points, which can hardly be handled by conventional machine learning approaches. To reduce the amount of data, pre-segmented VIS and FLU images were subdivided into a small number of regions ($N=[10,30]$) using k-means clustering of pixel colors. Consequently, plant and background image regions were compactly described by average colors of *N* k-means regions, or shortly AC-KMR, see Fig. 3. Thereby, the number of AC-KMR depends on variability of colors in image data. Juvenile homogeneously colored plants photographed against a uniform background require less AC-KMR than more color-rich adult plants and/or noisy background. By segmentation of images using plant type, age and camera view specific models, the same number of AC-KMR as defined in the model is used.

### Plant/background color region classification

**Fig. 5:**
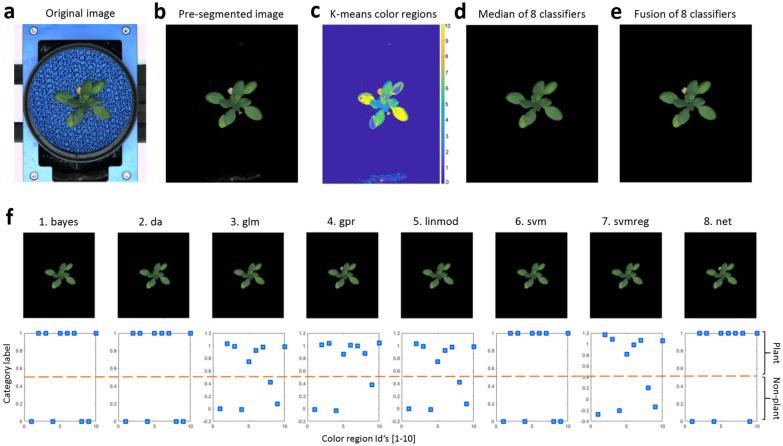
Classification between plant and non-plant (background) regions of pre-segmented image using pre-trained color classification models. **a** Original VIS image of mid-stage arabidopsis shoot in top-view. **b** Pre-segmented VIS image. **c** Clustering of (**b**) into 10 k-means color regions visualized with pseudo-colors. **d**, **e** Plant regions computed as median (**d**) or fusion (**e**) of eight binary classification results. **f** Plant regions predicted by eight color classification models. Plots show assignment of region labels to one of two binary color region categories: plant (1) or non-plant (0). The dotted-line indicates the threshold (tsh = 0.5) for assignment of fuzzy label estimates ($FE\in [0,1]$) of some classifiers (glm, gpr, linmod, svmreg) to one of two binary categories, i.e. if $FE\ge 0.5$ then plant (1), if $FE<0.5$ then non-plant (0)

Binary classification of background and plant regions is performed using average colors of *N* k-means regions (AC-KMR). From our experience, none of conventional classifiers showed exceptional performance throughout all plant species and age categories (as later discussed in details). Consequently, eight alternative classification models were trained to automatically separate AC-KMR using manually segmented and annotated data. Table 2 gives on overview of the eight binary models and the corresponding MATLAB (MathWorks, Inc.) functions that were used for supervised training and classification of plant and background regions. In the case of regression models (such as glm, grp, linmod, svmreg) that provide a ’fuzzy estimate’ ($FE\in [0,1]$) for category association, assignment to either plant (1) or background (0) category is performed using the fixed threshold 0.5, i.e. if $FE\ge 0.5$ then 1, if $FE<0.5$ then 0. In addition to predictions of eight distinctive classifiers, two additional segmentation results including median and fusion (i.e. logical OR) images of all eight classification models are computed, see Fig. 5.

**Table 2:** Overview of binary classification models and corresponding MATLAB functions trained using the input data *X* (i.e. 20 dimensional multi-color space representation of manually segmented FLU+VIS plant and background regions) and the vector of region labels *Y* (i.e. Y=0 for background, Y=1 for plant image regions)

#	Classification model	Acronym	MATLAB function
1	Naive Bayes model	bayes	fitcnb(X,Y)
2	Discriminant analysis	da	fitcdiscr(X,Y)
3	Generalized linear regression	glm	fitglm(X,Y)
4	Gaussian process regression	gpr	fitrgp(X,Y)
5	Linear model regression	linmod	fitlm(X,Y)
6	Binary support vector machine	svm	fitcsvm(X,Y)
7	Support vector machine regression	svm	fitrsvm(X,Y)
8	Neural network model	net	train(net,X,Y)
	(e.g., patternnet with N hidden layers)		net=patternnet(N)

### Small object removal

After application of the above segmentation steps images still may contain small artefacts (typically solitary objects) that have to be removed in order to avoid potential errors by subsequent calculation of phenotypic descriptors. Such small artefacts do not significantly affect the projection area, but can distort linear dimensions of segmented regions such as plant width, height, bounding box, convex hull, etc. Since growing plants exhibit different projection areas, one cannot rely on a fixed size threshold to remove such small artefacts,—separately segmented leaf tips of small plant shoots can be of the same size as small background structures. Consequently, a set of classification models based on eight classifiers from Table 2 was trained to detect small background (i.e. non-plant) structures on the basis of the object’s color ratios (R/B, R/G, G/B), size and vicinity to the largest image structure assessed by the Euclidean distance map using the *bwdist* MATLAB function. For decision making, the median values of object labels (i.e. plant or non-plant category) predicted by eight classifiers were used.

### Model evaluation measures

Accuracy of automated image segmentation was evaluated in terms of confusion matrix [TP FP; FN FP] (TP—true positive, FP—false positive, FN - false negative, TN - true negative) calculated on the basis of manually annotated and algorithmically predicted binary classification of k-means color-regions to either plant or background categories, and the overall model accuracy

1 $$\begin{aligned} A = \frac{TP+TN}{TP+FP+TN+FN} \in [0,1], \end{aligned}$$

which is closely related to other conventional measures, e.g., the Dice similarity coefficient (DSC). However, the accuracy of confusion matrix is more sensitive to failures in region classification than whole binary mask comparison using DSC.

### Model training and evaluation scenarios

Binary classification models for plant/background separation were trained and evaluated using several different scenarios. In particular, all eight classification models listed in Table 2 were separately trained for three different plant types (arabidopsis, wheat and maize), two different camera views (top/side view), three image modalities (FLU, VIS and FLU+VIS) and different plant developmental stages including: I—juvenile/small, II—mid-stage and III—adult/large shoots, as well as their combinations, i.e. I + II, I + III, II + III, I + II + III, resulting in totally 288 case-scenario models. The reason for such multiple model training is that optical appearance of plants and background regions significantly varies depending on screening facility, camera views, plant type and developmental stage. Consequently, it is not a-priori clear which of mostly linear models would be capable of accurately separating such highly variable and heterogeneous data. For evaluation of performance, trained models were tested on the same (training) data set as well as three new samples corresponding to juvenile, mid-stage and adult plants.

## Experimental results

FLU and VIS images of a totall of 80 arabidopsis, 526 maize and 564 wheat plants at different developmental stages were semi-automatically segmented using the kmSeg tool [15] into two (i.e. plant or background) categories as described above.

**Fig. 6:**
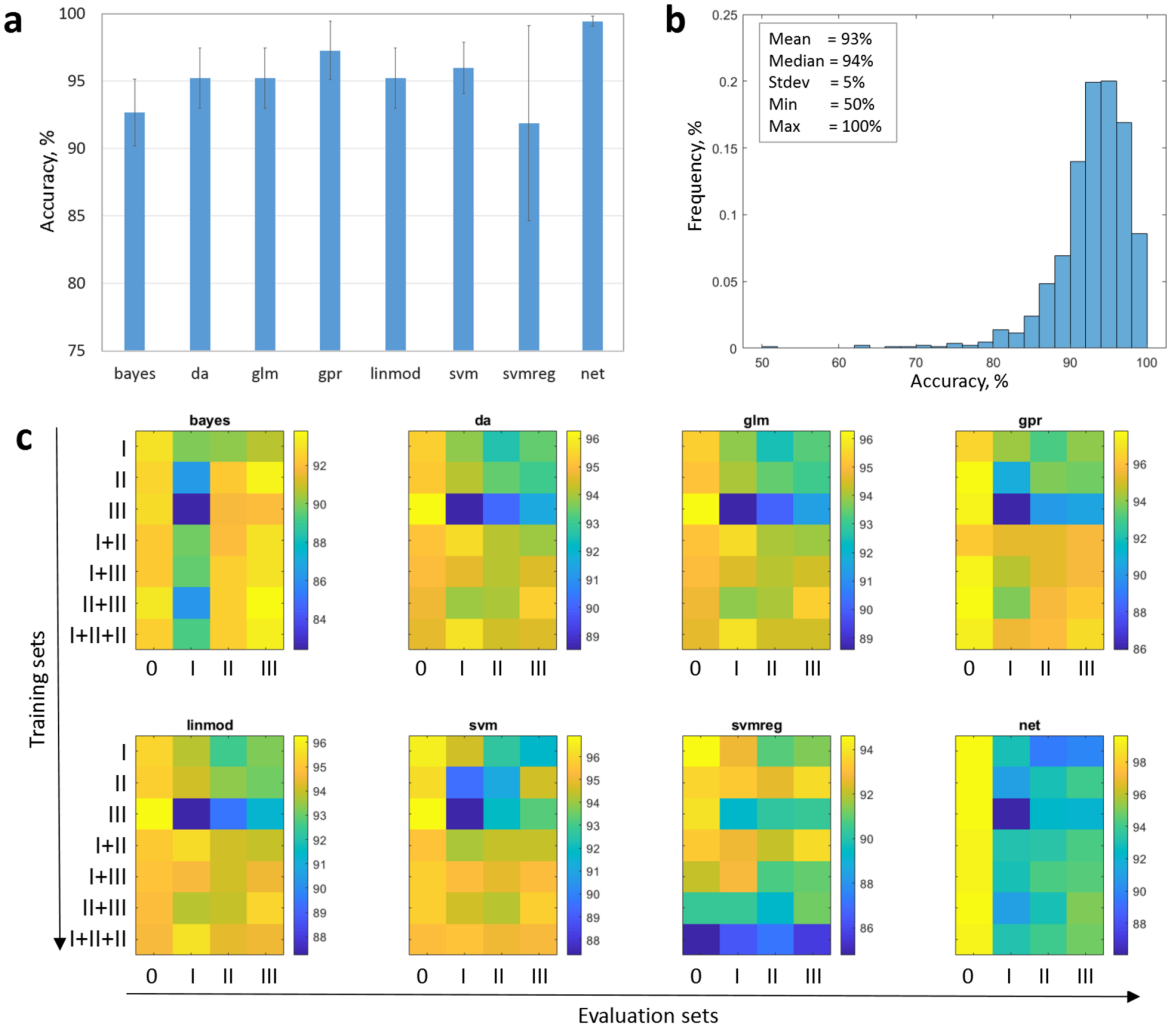
Summary of evaluation of eight different color classification models from Table 2. **a** Comparison of average model accuracy by reproducing the same training dataset they were trained to crossover different plant species and camera views. **b** Distribution of accuracy of all 288 case-scenario models considering for different camera views (top/side), plant types (arabidopsis, wheat, maize) and developmental stages by application to test samples. **c** Cross-evaluation of accuracy of classification models trained with manually annotated images of different plant developmental stages, including juvenile (I), mid-stage (II), adult (III) plants, and their combinations (i.e. I + II, I + III, II + III, I + II + III) by application to the same training set (0), as well as test samples of juvenile (I), mid-stage (II) and adult (III) plants

The registration-classification pipeline was applied to segment FLU/VIS image pairs of arabidopsis, wheat and maize images stepwise including (i) pre-segmentation of FLU/VIS images, (ii) automated co-registration of pre-segmented FLU/VIS images and (iii) classification of plant and non-plant structures in VIS image regions that were masked by the binary mask of the registered FLU image. As a consequence of different spatial resolutions and/or non-uniform leaf motion by relocation of plant from one photochamber to another, FLU/VIS alignment is not exact which manifests in inclusion of marginal background pixels, see Fig. 2. To remove marginal background regions in VIS images eight distinctive color models trained on all 288 case-scenarios including arabidopsis, wheat and maize plant/background appearance in different camera views and developmental stages were applied. Performance of all 288 models were evaluated in terms of confusion matrix between ground truth and predicted plant/background image regions by application to (i) the same (training) data set as well as three test samples corresponding to (ii) juvenile, (iii) mid- and (iv) adult stages of plant development. The results of all $1152=288*4$ tests including the confusion matrices and accuracy values can be found in Additional file 1: Table S1. A brief summary of the model performance is shown in Fig. 6. As one can see in Fig. 6a, all eight classification models are capable to reproduce the same training data they were trained to with an average accuracy of more than $90\%$. Fig. 6b shows the distribution of accuracy crossover all 288 classification models, which exhibits following cumulative statistics:

$$\begin{aligned} \text {Mean}&= 93\%\\ \text {Median}&= 94\%\\ \text {SD}&= 5\%\\ \text {Min}&= 50\%\\ \text {Max}&= 100\% \end{aligned}$$

The best and worst performers by reproduction of the training data are net and svmreg, respectively. The outperformance of the non-linear neural net over other linear models by the self-reproduction test is not surprising. However, by application to other test samples the net model does not appear to be advantageous in comparison to linear models, see Fig. 6c. Furthermore, it is evident that all models that were trained with image data of adult plants (III) show significantly poorer performance by application to juvenile plant species (I). This fact can be traced back to significant differences between color signatures of juvenile (typically light green) and adult (rather dark green and sometimes even yellow and/or red) plant leaves. With exception of svmreg, most classification models show the best performance crossover plant species of different developmental stages when they were trained on mixed datasets combining juvenile, mid-stage and adult plants (I + II + III).

The whole pipeline of registration-classification based segmentation (RCS) of multimodal greenhouse plant images is provided as an executable command-line tool

*rcs.exe* <*input imgages*> <*output images*> <*class model*> <*opts*>

suitable for script integration and high-context image processing from our homepage https://ag-ba.ipk-gatersleben.de/rcs.html. For the given pair of unregistered and unsegmented fluorescent and visible light images as well as reference FLU/VIS images, the RCS tool performs automated registration-classification based segmentation and writes out registered and segmented FLU/VIS images as well as further optional files in output. If users do not have reference images, they can generate them on their own under consideration of a typical background color in the plant containing images, for example,—black FLU and light gray VIS reference images of the same size as plant containing FLU/VIS images. A detailed description the RCS tool can be found in the user guide available with the above file repository.

## Discussion

Segmentation of a large amount of multimodal image data from greenhouse phenotyping experiments is the first challenging step of any image analysis pipeline aiming at quantitative plant phenotyping. However, straightforward segmentation of some image modalities including wide-spread visible light images is hampered by a number of natural and technical factors including inhomogeneous illumination of photochambers, dynamic optical appearance of developing plants, shadows, reflections and occlusion in plant and background regions. To overcome the limitations of unimodal image analysis, an approach to plant image segmentation based on multimodal image registration followed by classification of plant and marginal background regions was developed. Our experimental results using eight conventional classifiers and totally 288 case-scenario models considering different camera views, plant types and developmental stages demonstrate that plant segmentation with the average accuracy of $93\%$ (SD=$5\%$) crossover all tested models can be achieved. More accurate segmentation can be performed using suitable case-scenario models including FLU, VIS as well as combined FLU/VIS based classification of plant and marginal background regions. Furthermore, our evaluation studies show that classifiers trained on a mixed image data including different plant developmental stages and optical appearance outperform classification models tailored to a narrow plant phenotype. Despite a broad spectrum of optical case-scenarios our classification models are based on optical setups of our particular three screening platforms that exhibit light background regions in contrast to darker plant structures. In case of strongly deviating optical conditions and/or plant appearance retraining of classification models should be taken into consideration.

## Conclusion

Highly variable optical appearance of different plant and background structures makes segmentation of greenhouse images a non-trivial task. To overcome shortcomings of unimodal image analysis, here we suggest a two-step registration-classification approach which reduces complexity of whole image segmentation to classification of pre-segmented fluorescent and visible light plant and marginal background image regions. Our experimental results demonstrate that this approach enables segmentation of different plant types in different developmental stages from different camera view with sufficiently high accuracy suitable for application in fully automated high-throughput manner. A command-line tool provided with this work enables quantitative plant researchers to efficiently integrate our registration-classification based image segmentation algorithms in custom image processing pipelines.

## Supplementary information

**Additional file 1: Table S1.** Summary of evaluation of 288 case-scenario models for plant image segmentation against ground truth data. 288 case-scenarios result from training of eight classification models (i.e. bayes, da, glm, gpr, linmod, svm, svmreg, net) for three different plant types (arabidopsis, wheat and maize), two different camera views (top/side view), three image modalities (FLU, VIS and FLU+VIS) and three plant developmental stages including: I—juvenile/small, II—mid-stage and III—adult/large shoots, as well as their combinations, i.e. I + II, I + III, II + III, I + II + III.

## Data Availability

Examples of data used and analyzed in this study are provided in supplementary materials. Further datasets are available from the corresponding author on request.
